# Development and validation of a machine learning model and nomogram for predicting brain metastasis in lung cancer: a population-based study

**DOI:** 10.1016/j.clinsp.2025.100843

**Published:** 2025-11-13

**Authors:** Yukai Zeng, Fengwu Lin, Lening Zhang, Jingyuan Jiang, Chen Zhang, Dongliang Qin, Zhe Zhang

**Affiliations:** Department of Thoracic Surgery, China-Japan Union Hospital of Jilin University, Changchun, China

**Keywords:** Brain metastasis, Lung cancer, Machine learning, Online Calculator, Nomogram

## Abstract

**Objective:**

In advanced-stage lung cancer management, the brain emerges as the predominant metastatic site. Accurately forecasting both the likelihood of intracranial metastasis and patient survival duration has become a critical clinical priority, though existing predictive systems demonstrate insufficient precision.

**Materials and method:**

This study conducted a retrospective cohort study utilizing SEER database records of lung cancer cases spanning 2010‒2015, incorporating demographic and clinical data. Multivariate logistic regression was initially employed for variable selection, followed by Cox proportional hazards modeling to identify risk factors associated with brain metastasis development and prognostic indicators. Significant predictors were subsequently integrated into six machine learning algorithms to construct risk stratification frameworks. Model performance underwent comprehensive evaluation through area under the ROC curve metrics, classification error matrices, and calibration curve assessments.

**Results:**

This investigation analyzed a cohort of 25,072 lung cancer patients. Multiple clinical parameters including demographic characteristics (age, gender, ethnicity), tumor features (primary location, histological classification, differentiation grade), disease progression markers (T/N staging), therapeutic interventions (surgical procedures, radiotherapy, chemotherapy), and social factors (marital status) emerged as significant predictors of prognosis. Metastasis risk analysis identified age, tumor grade, histopathological type, and treatment modalities as key determinants. Comparative evaluation of predictive models revealed that the Gradient Boosting Machine (GBM) algorithm demonstrated superior performance in both cross-validation and internal testing phases.

**Conclusion:**

The developed GBM-based predictive system offers clinicians a user-friendly web application for estimating cerebral metastasis probability in lung cancer cases, enhancing personalized treatment planning through accessible risk stratification.

## Highlights

• Brain metastasis focus: Predicts brain spread and survival in lung cancer.
• Big-data study: 25 k SEER patients, Cox/logistic regressions.
• Best ML: GBM wins internal/external tests.
• Clinician web tool: One-click risk calculator online.
• Nomogram: Simple chart forecasts prognosis.

## Introduction

Lung carcinoma remains the primary contributor to cancer-related mortality worldwide, with cerebral metastases emerging as a frequent complication that substantially diminishes survival outcomes. Current prognostic tools for predicting brain metastasis development exhibit restricted clinical utility due to methodological constraints. These models frequently depend on traditional statistical methodologies, employ insufficient sample cohorts, and incorporate potentially biased variable selection processes. This investigation aims to construct an innovative predictive framework integrating machine learning-enhanced nomographic analysis with cloud-based computational tools. By leveraging advanced pattern recognition capabilities, this approach seeks to improve prognostic precision while offering clinicians an accessible decision-support system for personalized therapeutic planning and patient counseling.

Lung cancer remains a global health burden due to its substantial disease prevalence and fatality rates.^1^ Statistical analyses reveal an annual incidence of roughly 53.6 cases per 100,000 population, with corresponding mortality figures reaching 45.6 per 100,000 individuals.^2^ Histologically, this malignancy is classified into two main pathological subtypes: Non-Small Cell Lung Carcinoma (NSCLC) and Small Cell Lung Carcinoma (SCLC). NSCLC represents the predominant form, accounting for 85 %‒90 % of diagnoses, with further subdivisions including adenocarcinoma, squamous cell carcinoma, and large cell carcinoma variants.^3^ The diagnostic challenge arises from the absence of distinct clinical manifestations in initial disease stages, frequently leading to delayed detection until advanced progression with distant metastatic spread. Metastatic patterns typically emerge through three distinct mechanisms: lymphatic system involvement, bloodstream dissemination, and contiguous tissue infiltration. Common metastatic sites include the brain, skeletal system, hepatic tissue, and adrenal glands, with these secondary growths significantly influencing treatment outcomes and survival probabilities.

The central nervous system has been recognized as a predominant metastatic target in pulmonary carcinoma cases.^4^, ^5^, ^6^ Clinical data indicate that nearly half of all lung cancer patients eventually experience intracranial spread (Brain Metastasis, BM). The emergence of neurological dissemination events correlates with diminished survival prospects, demonstrating median life expectancy ranging from 3‒6 months and posing substantial therapeutic difficulties.^7^^,^^8^ Furthermore, cerebral metastases in pulmonary malignancies markedly influence clinical outcomes, reflecting terminal disease progression and indicating unfavorable survival trajectories. These pathological developments may precipitate neurovascular complications accompanied by clinical manifestations including chronic discomfort, cachexia, and potential emergence of Multi-Organ Failure Syndromes (MODS) in terminal stages. This investigation utilized cutting-edge machine learning algorithms to perform systematic analysis on patients with pulmonary carcinoma-related cerebral metastases. Artificial intelligence-driven analytical approaches demonstrate enhanced capacity for integrating multi-layered data patterns when processing extensive clinical datasets, thereby substantially improving predictive model precision.

Machine learning demonstrates superior predictive capabilities when contrasted with conventional logistic regression approaches. By employing sophisticated computational frameworks and large-scale data analytics, this methodology enhances the accuracy of predictive modeling and analytical outcomes.^9^ The application of machine learning spans multiple sectors, from self-driving vehicle systems to intricate decision-making scenarios in complex systems, highlighting its exceptional computational efficiency and adaptive nature.^10^ Within biomedical research, the exponential growth of healthcare data repositories has created novel opportunities for advancing disease understanding and health optimization. These advanced algorithms possess the dual capacity to systematically process vast medical datasets while identifying clinically relevant patterns, thereby accelerating progress in tailored medical interventions and individualized care strategies.^11^^,^^12^ This technological advancement has demonstrated practical utility across various medical applications, including diagnostic procedures, therapeutic interventions, and patient monitoring systems.

The advancements in this field have facilitated earlier diagnosis rates, more personalized therapeutic strategies, and improved overall disease management for oncology patients.^13^ Given the differences in clinicopathological characteristics among individuals with pulmonary malignancies, diverse treatment approaches and survival expectations exist, resulting in considerable variability in clinical outcomes. However, current studies focusing on cerebral metastases from primary lung tumors remain scarce, creating clinical challenges for medical practitioners when formulating intervention protocols. This investigation, therefore, aims to develop and validate advanced machine learning frameworks combined with prognostic nomogram systems ‒ specifically those demonstrating superior predictive accuracy ‒ for assessing cerebral metastasis risks and predicting survival timelines in lung cancer cases.

## Materials and methods

### Patient cohort

The Surveillance, Epidemiology, and End Results (SEER) Program [www.seer.cancer.gov] provided data through its SEER*Stat tool (version 8.4.1), accessing the Incidence-SEER Research Dataset covering 17 registries. This population-based registry contained time-dependent county attributes from 1990‒2022, including socioeconomic and geographic indicators, with case information spanning 2000‒2021. The National Cancer Institute's Division of Cancer Control and Population Sciences (DCCPS) released the analyzed dataset in April 2024, utilizing November 2023 submissions. The study cohort comprised individuals diagnosed with primary lung malignancies (encompassing small cell carcinoma, squamous cell carcinoma, large cell carcinoma, and adenocarcinoma) during 2010‒2015. Case selection criteria incorporated multiple parameters: Anatomic site classification using ICD-O-3/WHO 2008 guidelines (“Lung and Bronchus”), malignant behavior designation (ICD-O-3 code/3), and restriction to first-primary tumors only.

Histology classifications (ICD-O-3) included small cell lung carcinoma (8041/3), squamous cell lung carcinoma (8070/3), large cell lung carcinoma (8012/3), and pulmonary adenocarcinoma (8140/3). All histological categorizations aligned with SEER's “Site-Histology Recode for Lung and Bronchus” (2010–2015 edition) to maintain database compatibility. Exclusion parameters comprised: 1) Omission of patients lacking survival duration data 2) Removal of individuals with undocumented follow-up status; 3) Elimination of subjects without brain metastasis documentation; 4) Exclusion of cases missing AJCC T/N stage specifications; 5) Disqualification of records lacking racial background, histological verification, or tumor differentiation details; 6) Exclusion of patients with undetermined primary tumor location or laterality information; 7) Removal of cases without recorded therapeutic interventions.

**Fig. 1:**
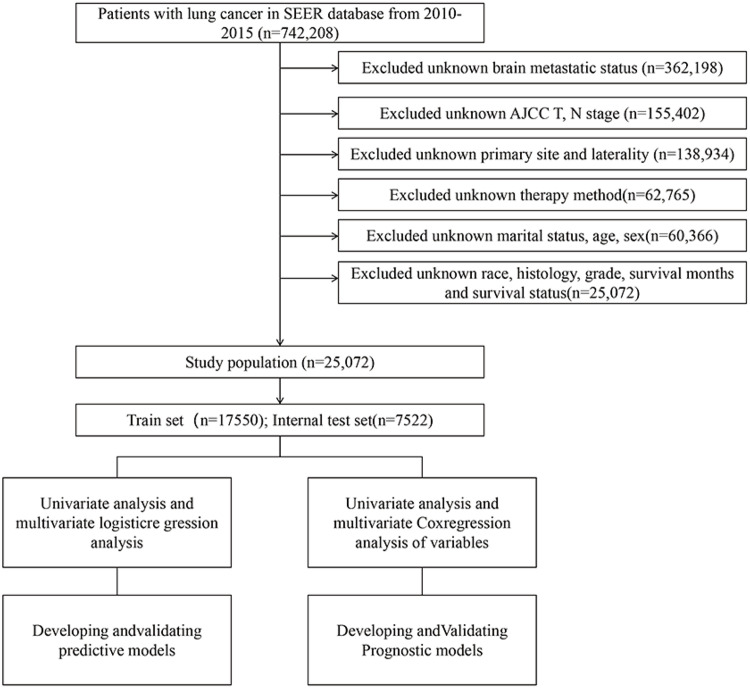
The study flow chart of case screening.

Cases lacking documented marital status, age, or gender information were omitted from the analysis. The case selection procedure is visually represented in Fig. 1, with comprehensive predictor details available in Supplementary Table 2. Given the SEER database's constraint of offering cross-sectional patient data (lacking temporal treatment records such as therapeutic interventions and BM detection timelines), the precise sequence between therapeutic modalities (surgical procedures, radiotherapy, chemotherapeutic regimens) and BM development remains undetermined - a recognized limitation in SEER-related investigations. Following established research protocols, these therapeutic variables were incorporated as predictive factors, with 'brain metastasis' in the OS prognostic model characterized as the initial presentation status during lung cancer identification. Regarding principal predictors (surgical intervention, radiotherapy), these parameters were operationally defined as primary therapeutic strategies determined post-baseline staging, aligning with clinical protocols and SEER research methodologies.

### Data screening

This investigation incorporated fourteen distinct parameters associated with clinicopathological profiles and demographic attributes for comprehensive evaluation. The demographic subset encompassed four key factors: chronological age, biological sex, marital status categorization, and ethnic background classification. The clinicopathological parameters analyzed encompassed primary tumor location, laterality, histological classification, tumor grade, T-category, N category, surgical intervention, radiotherapy administration, chemotherapy regimen, and presence of brain metastases. Lung cancer histotypes were categorized using ICD-O-3 codes into adenocarcinoma, squamous cell carcinoma, large cell carcinoma, and small cell carcinoma subtypes. All malignancies were initially classified according to AJCC 7th edition criteria (aligning with SEER's 2010‒2015 documentation standards) and later reclassified following AJCC 9th edition guidelines through official conversion protocols and SEER staging parameters. Optimal age thresholds were established through X-tile software (v3.6.1) analysis,^14^ with statistical comparisons performed using chi-square tests. Fourteen prognostic factors were evaluated across three domains: Demographic factors (age at diagnosis, gender, ethnicity, marital status), tumor characteristics (primary site, laterality, histopathological type, differentiation grade, T-stage, N-stage), along therapeutic interventions (surgical resection, radiation therapy, systemic chemotherapy). Outcome-related predictors (including baseline cerebral metastases status) were established prior to analytical procedures, with comprehensive criteria outlined in Supplementary Table 2. A complete-case analytical approach was utilized to eliminate subjects containing undetermined values for these predictive factors, achieving a 0 % missing data rate across all parameters within the finalized study population of 25,072 participants.

### Data preprocessing

Statistical analyses in this investigation were executed through Python (3.11.3), SPSS 27, and R software (4.4.1). The research utilized SPSS for performing both logistic and Cox regression analyses, serving dual purposes of identifying predictive variables for machine learning model construction and prognostic factor evaluation in survival analysis. Preliminary univariate logistic regression identified potential predictors of cerebral metastasis in lung cancer cases (statistically significant at *p* < 0.05), which subsequently underwent multivariate analysis. Variables demonstrating persistent significance (*p* < 0.05) were selected for integration into advanced machine learning frameworks. Parallel analytical procedures using Cox proportional hazards models revealed prognostic determinants (*p* < 0.05), subsequently visualized through a prognostic nomogram. Inter-variable relationships were systematically examined through comprehensive correlation assessments. To address class imbalance, this approach prioritizes augmenting the sample quantity of underrepresented categories to enhance predictive performance. Feature significance was assessed through the Permutation Importance framework integrated within the algorithmic framework.^15^^,^^16^ The analytical process involved importing 'dplyr' and 'caret' libraries in the R environment, followed by randomized allocation of SEER database records into training and internal validation cohorts with a 70:30 distribution ratio. Discrepancy detection between datasets was conducted through chi-square testing.

### ML model establishment and evaluation

Six distinct classification methodologies were implemented, combining three ensemble approaches (RF, GBM, XGB) with three fundamental classifiers (LR, DT, NBC).^17^ Model development was implemented in a Python programming environment, incorporating rigorous training protocols for each algorithm. The SEER dataset underwent division into ten cross-validation folds within the training cohort.^18^ To address data imbalance, researchers implemented the Synthetic Minority Oversampling Technique (SMOTE) for preprocessing, with this augmentation strategy exclusively applied to the internal validation subset.^19^ All six computational models (RF, XGB, GBM, LR, DT, NB) maintained standardized parameters (random_state fixed at 123 for experimental consistency). The assessment framework expanded beyond conventional metrics to include Precision-Recall AUC (PR-AUC), Positive Predictive Value (PPV), and Negative Predictive Value (NPV) for comprehensive model evaluation. The present study’s research team developed calibration curves for the six classifiers using the internal testing cohort. During internal validation, raw test data underwent direct processing through the established predictive models. Algorithm performance analysis incorporated multiple quantitative measures, including receiver operating characteristic curves and associated statistical parameters. The model's performance was evaluated using key metrics, including the area under the receiver operating characteristic curve (AUC), along with sensitivity, specificity, classification accuracy, and F-measure scores. Clinical relevance was examined through probability density visualization and clinical utility curve analysis. Building upon the optimal model configuration, the authors designed an interactive web application for clinical prediction. Detailed algorithmic configurations and hyperparameter settings are comprehensively documented in Supplementary Table 3, with established default values ensuring methodological consistency and reproducibility ‒ these baseline configurations align with published performance benchmarks and represent standard initialization practices in SEER-related pulmonary oncology research. The complete analytical framework and SEER*Stat query specifications represent collaborative intellectual assets protected under institutional knowledge-sharing agreements. For replication purposes, researchers may contact the corresponding investigator (Zhe Zhang; E-mail: zz9412182021@163.com) to request access to these computational resources under appropriate data-sharing protocols.

### Development and construction of predictive model

The statistical analysis employs R software (version 4.4.1) with integrated 'rms', 'foreign', 'survival', and 'survivalROC' packages for nomogram creation. Additionally, the authors evaluate predictive performance using multiple validation metrics, including concordance index, receiver operating characteristic analysis, calibration curves, decision curve evaluation, and discrimination enhancement scores through IDI assessment.

## Results

### Demographic and clinical profiles of study participants

The study cohort comprised 25,072 lung cancer cases retrieved from the SEER database, stratified into training (70 %) and internal validation (30 %) cohorts through random allocation. Demographic and clinical characteristics evaluated included age at diagnosis, gender, ethnicity, tumor location, lateralization, differentiation status, histological subtype, TNM classification components (T and N stages), therapeutic interventions (surgical resection, radiotherapy, chemotherapy), marital status, and presence of cerebral metastases. Intergroup variations were assessed through chi-square testing, revealing comparable distributions across all parameters (*p* > 0.05; see Supplementary Table 1). Cerebral metastases prevalence showed minimal variation between cohorts ‒ 795 cases (4.5 %) in the training group versus 361 cases (4.8 %) in the validation group (detailed in Table 1).

**Table 1:** Clinical and pathological characteristics of the train set and internal test set.

Variables	Train Set (n = 17,552)		Internal Test Set (n = 7520)	
Age				
< 70	7375	42.0 %	3214	42.7 %
70‒79	7858	44.8 %	3342	44.4 %
> 79	2317	13.2 %	966	12.8 %
Sex				
Female	9226	52.6 %	3937	52.3 %
Male	8324	47.4 %	3585	47.7 %
Race				
White	14,187	80.8 %	6051	80.4 %
Black	1696	9.7 %	762	10.1 %
Other	1667	9.5 %	709	9.4 %
Primary Site				
Upper lobe	10,691	60.9 %	4601	61.2 %
Lower lobe	5687	32.4 %	2421	32.2 %
Middle lobe	831	4.7 %	357	4.7 %
Main bronchus	341	1.9 %	143	1.9 %
Laterality				
Left	7376	42.0 %	3089	41.1 %
Right	10,174	58.0 %	4433	58.9 %
Grade				
Grade I	2506	14.3 %	1101	14.6 %
Grade II	8271	47.1 %	3456	45.9 %
Grade III	6456	36.8 %	2821	37.5 %
Grade IV	317	1.8 %	144	1.9 %
Histology				
Adenocarcinoma	10,720	61.1 %	4649	61.8 %
Squamous carcinoma	5964	34.0 %	2487	33.1 %
Small cell carcinoma	643	3.7 %	268	3.6 %
Large cell carcinoma	223	1.3 %	118	1.6 %
T				
T1	7690	43.8 %	3284	43.7 %
T2	4876	27.8 %	2048	27.2 %
T3	2476	14.1 %	1069	14.2 %
T4	2508	14.3 %	1121	14.9 %
N				
N0	11,877	67.7 %	5062	67.3 %
N1	1726	9.8 %	753	10.0 %
N2	3185	18.1 %	1360	18.1 %
N3	762	4.3 %	347	4.6 %
Surgery				
No	7193	41.0 %	3092	41.1 %
Yes	10,357	59.0 %	4430	58.9 %
Radiation				
No	11,532	65.7 %	4889	65.0 %
Yes	6018	34.3 %	2633	35.0 %
Chemotherapy				
No	11,404	65.0 %	4908	65.2 %
Yes	6146	35.0 %	2614	34.8 %
Marriage				
Single	8378	47.7 %	3513	46.7 %
Married	9172	52.3 %	4009	53.3 %
Brain metastasis				
No	16,755	95.5 %	7161	95.2 %
Yes	795	4.5 %	361	4.8 %

### Univariate and multivariate logistic regression analyses

This study utilized univariate and multivariate logistic regression methods to identify eight clinical predictors associated with cerebral metastasis development. Key variables encompassed patient age, tumor grade, histological type, tumor staging (T and N classifications), along with treatment modalities including surgical intervention, radiotherapy, and chemotherapy (statistical significance *p* < 0.05, see Table 2). Building upon these predictors, six predictive models were developed through machine learning techniques.

**Table 2:** Univariate analysis and multivariate logistic regression analysis of variables.

Variables	Univariate Logistic Analysis		Multivariate Logistic Analysis	
	OR (95 % CI)	p-value	OR (95 % CI)	p-value
Age				
< 68	Reference		Reference	
68‒79	0.624 (0.536‒0.728)	<0.001	0.640 (0.542‒0.757)	<0.001
> 79	0.592 (0.465‒0.755)	<0.001	0.434 (0.333‒0.566)	<0.001
Sex				
Female	Reference			
Male	1.071 (0.929‒1.234)	0.347		
Race				
White	Reference			
Black	1.514 (1.225‒1.872)	<0.001		
Other	1.193 (0.944‒1.508)	0.139		
Primary Site				
Upper lobe	Reference			
Lower lobe	0.814 (0.693‒0.957)	0.012		
Middle lobe	0.881 (0.621‒1.251)	0.480		
Main bronchus	1.934 (1.316‒2.842)	0.001		
Laterality				
Left	Reference			
Right	1.039 (0.900‒1.201)	0.600		
Grade				
Grade I	Reference		Reference	
Grade II	1.935 (1.329‒2.818)	0.001	1.959 (1.327‒2.893)	0.001
Grade III	6.758 (4.715‒9.688)	<0.001	3.649 (2.497‒5.334)	<0.001
Grade IV	11.808 (7.332‒19.015)	<0.001	4.548 (2.550‒8.112)	<0.001
Histology				
Adenocarcinoma	Reference		Reference	
Squamous carcinoma	0.434 (0.360‒0.524)	<0.001	0.198 (0.162‒0.241)	<0.001
Small cell carcinoma	2.495 (1.940‒3.209)	<0.001	0.597 (0.426‒0.837)	0.003
Large cell carcinoma	1.303 (0.767‒2.216)	0.328	0.683 (0.384‒1.214)	0.194
T				
T1	Reference		Reference	
T2	2.692 (2.171‒3.338)	<0.001	2.390 (1.897‒3.012)	<0.001
T3	3.783 (2.996‒4.777)	<0.001	2.270 (1.753‒2.938)	<0.001
T4	6.707 (5.432‒8.279)	<0.001	2.905 (2.283‒3.696)	<0.001
N				
N0	Reference		Reference	
N1	2.592 (2.054‒3.270)	<0.001	1.942 (1.492‒2.528)	<0.001
N2	4.466 (3.781‒5.274)	<0.001	1.512 (1.238‒1.848)	<0.001
N3	6.306 (4.958‒8.020)	<0.001	1.557 (1.181‒2.052)	0.002
Surgery				
No	Reference		Reference	
Yes	0.064 (0.050‒0.081)	<0.001	0.120 (0.090‒0.159)	<0.001
Radiation				
No	Reference		Reference	
Yes	7.511 (6.329‒8.913)	<0.001	2.723 (2.209‒3.356)	<0.001
Chemotherapy				
No	Reference		Reference	
Yes	2.717 (2.351‒3.140)	<0.001	0.687 (0.565‒0.834)	<0.001
Marriage				
Unmarride	Reference			
Married	0.884 (0.766‒1.019)	0.088		

### Univariable and multivariable cox regression analysis

To enhance the prognostic nomogram's accuracy, both univariate and multivariate Cox regression analyses were implemented. This analytical approach revealed thirteen clinically significant variables impacting overall survival outcomes. The prognostic indicators comprised demographic factors (age, gender, ethnicity, marital status), tumor characteristics (primary location, histopathology, differentiation grade, TNM staging, b**rain metastasis**), therapeutic interventions (surgical management, radiation therapy, chemotherapeutic treatment), and metastatic status (*p* < 0.05 as detailed in Table 3).

**Table 3:** Univariate analysis and multivariate Cox regression analysis of variables.

Variables	Univariate Cox Analysis		Multivariate Cox Analysis	
	HR (95 % CI)	p-value	HR (95 % CI)	p-value
Age				
< 68	Reference		Reference	
68‒79	1.362 (1.279‒1.451)	<0.001	1.402 (1.314‒1.496)	<0.001
> 79	2.291 (2.118‒2.477)	<0.001	1.779 (1.637‒1.933)	<0.001
Sex				
Female	Reference		Reference	
Male	1.580 (1.494‒1.671)	<0.001	1.369 (1.291‒1.452)	<0.001
Race				
White	Reference		Reference	
Black	1.081 (0.988‒1.183)	0.088	0.941 (0.859‒1.030)	0.189
Other	0.699 (0.626‒0.780)	<0.001	0.745 (0.666‒0.832)	<0.001
Primary Site				
Upper lobe	Reference		Reference	
Lower lobe	0.973 (0.915‒1.034)	0.375	1.086 (1.021‒1.156)	0.009
Middle lobe	0.951 (0.831‒1.090)	0.472	1.087 (0.948‒1.245)	0.231
Main bronchus	3.147 (2.739‒3.617)	<0.001	1.534 (1.330‒1.769)	<0.001
Laterality				
Left	Reference			
Right	0.989 (0.935‒1.047)	0.710		
Grade				
Grade I	Reference		Reference	
Grade II	1.900 (1.697‒2.127)	<0.001	1.351 (1.202‒1.518)	<0.001
Grade III	3.212 (2.873‒3.591)	<0.001	1.550 (1.378‒1.744)	<0.001
Grade IV	4.916 (4.062‒5.948)	<0.001	1.751 (1.392‒2.202)	<0.001
Histology				
adenocarcinoma	Reference		Reference	
Squamous carcinoma	2.196 (2.072‒2.328)	<0.001	1.418 (1.331‒1.511)	<0.001
Small cell carcinoma	3.326 (2.960‒3.738)	<0.001	1.581 (1.366‒1.830)	<0.001
Large cell carcinoma	1.943 (1.557‒2.425)	<0.001	1.605 (1.281‒2.010)	<0.001
T				
T1	Reference		Reference	
T2	1.889 (1.750‒2.039)	<0.001	1.570 (1.451‒1.700)	<0.001
T3	2.993 (2.753‒3.255)	<0.001	1.988 (1.816‒2.177)	<0.001
T4	4.743 (4.392‒5.122)	<0.001	2.311 (2.114‒2.527)	<0.001
N				
N0	Reference		Reference	
N1	1.699 (1.549‒1.863)	<0.001	1.563 (1.417‒1.725)	<0.001
N2	3.054 (2.866‒3.255)	<0.001	1.635 (1.514‒1.765)	<0.001
N3	3.655 (3.295‒4.055)	<0.001	1.561 (1.392‒1.751)	<0.001
Surgery				
NO	Reference		Reference	
Yes	0.182 (0.171‒0.194)	<0.001	0.201 (0.185‒0.219)	<0.001
Radiation				
No	Reference		Reference	
Yes	2.022 (1.912‒2.137)	<0.001	0.540 (0.503‒0.580)	<0.001
Chemotherapy				
No	Reference		Reference	
Yes	1.360 (1.285‒1.439)	<0.001	0.596 (0.555‒0.641)	<0.001
Marriage				
Unmarride	Reference		Reference	
Married	0.750 (0.709‒0.793)	<0.001	0.858 (0.809‒0.909)	<0.001
Brain metastasis				
No	Reference		Reference	
Yes	3.575 (3.257‒3.922)	<0.001	2.224 (2.012‒2.458)	<0.001

### Feature correlation and significance evaluation

**Fig. 2:**
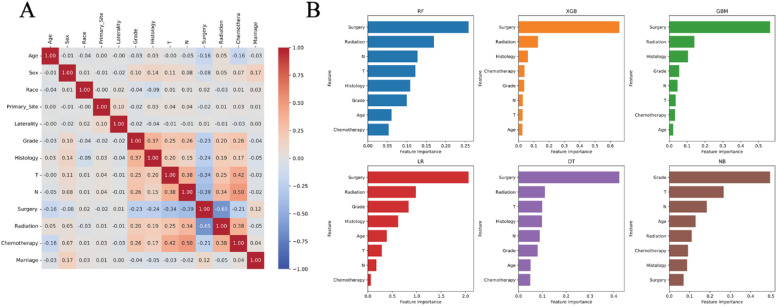
(A) Heat map of the correlation of features. (B) Feature importance of different models.

Statistical evaluation of variable relationships was conducted using Spearman's correlation method to examine interdependencies within the dataset. The visualized correlation matrix (Fig. 2A) demonstrated moderate-strength associations among the thirteen examined parameters, with coefficients ranging between ±0.5. This pattern suggests minimal interrelatedness among the analyzed variables. Feature importance assessments across multiple analytical approaches, as shown in Fig. 2B, revealed that parameters identified through both single-variable and multi-factor regression approaches showed significant predictive performance in all six computational frameworks. Notably, surgical procedures maintained consistent prominence as the most influential parameter across predictive models, underscoring their critical association with cerebral metastasis development in pulmonary carcinoma cases. The majority of algorithmic approaches confirmed this pattern, Surgical intervention emerges as the primary determinant within the modeling framework, with radiotherapy demonstrating substantial secondary impact. Other variables exhibit differential significance across alternative analytical approaches.

### Model performance

**Fig. 3:**
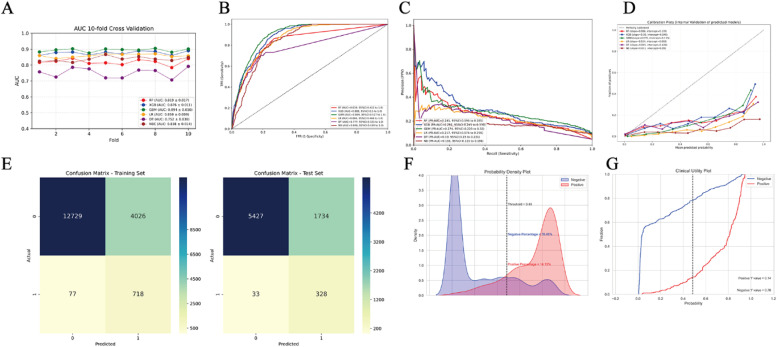
(A) Ten-fold cross-validation results of different machine learning models. (B) The ROC (Receiver Operating Characteristic) curves of different machine learning models in internal test set. (C) The PR-AUC (Precision-Recall Area Under the Curve) of different machine learning models in internal test set. (D) The calibration charts of different machine learning models in internal test set. (E) The confusion matrix of the GBM (Gradient Boosting Machine) model in the train set and the internal test set. TP, True Positive; TN, True Negative; FP, False Positive; FN, False Negative. (F) Probability density plot of gradient boosting machine model. (G) The clinical impact curve of the GBM (Gradient Boosting Machine) model.

The performance metrics of six predictive algorithms are visually represented in Fig. 3A‒D and Table 4. Analysis of internal 10-fold cross-validation results (Fig. 3A) revealed the Gradient Boosting Machine (GBM) algorithm demonstrated superior predictive capability compared to other models, attaining a mean AUC of 0.894, while the XGBoost (XGB) model showed competitive performance with an AUC of 0.876. Subsequent validation testing outcomes detailed in Table 4 and Fig. 3B‒D confirm the sustained advantage of the GBM model, achieving peak AUC performance (0.899), complemented by balanced metrics including 0.852 accuracy, 0.729 sensitivity, and 0.858 specificity. The model exhibited a PR-AUC of 0.274 alongside predictive values of 0.137 PPV and 0.996 NPV (Table 4). In clinical applications for detecting lung cancer-related Brain Metastases (BM) ‒ particularly crucial given the low observed BM prevalence (4 %‒5 %) ‒ the framework prioritizes diagnostic reliability by emphasizing the critical need to minimize false-negative classifications, thereby ensuring timely therapeutic interventions for high-risk cohorts.

**Table 4:** Prediction performance of different models.

Model	RF	XGB	GBM	LR	DT	NB
AUC	0.839	0.888	0.899	0.866	0.777	0.846
AUC_95 % CI	0.422‒1.000	0.500‒1.000	0.517‒1.000	0.466‒1.00	0.325‒1.000	0.434‒1.000
PR_AUC	0.241	0.292	0.274	0.217	0.190	0.159
PR_AUC_95 % CI	0.196‒0.285	0.245‒0.339	0.228‒0.320	0.174‒0.259	0.15‒0.231	0.121‒0.196
Accuracy	0.904	0.909	0.852	0.743	0.903	0.738
Accuracy_95 % CI	0.891‒0.917	0.896‒0.922	0.836‒0.867	0.724‒0.763	0.89‒0.916	0.718‒0.757
Sensitivity	0.441	0.510	0.729	0.817	0.427	0.830
Sensitivity_95 % CI	0.341‒0.541	0.409‒0.610	0.64‒0.818	0.739‒0.894	0.328‒0.527	0.755‒0.905
Specificity	0.928	0.929	0.858	0.739	0.927	0.733
Specificity_95 % CI	0.916‒0.939	0.918‒0.941	0.842‒0.874	0.72‒0.759	0.915‒0.939	0.713‒0.753
PPV	0.186	0.185	0.137	0.100	0.180	0.116
PPV_95 % CI	0.144‒0.230	0.146‒0.226	0.111‒0.164	0.081‒0.12	0.138‒0.224	0.093‒0.14
NPV	0.978	0.983	0.996	0.998	0.977	0.991
NPV_95 % CI	0.971‒0.985	0.976‒0.989	0.992‒0.999	0.995‒1.0	0.969‒0.984	0.985‒0.996
F-score	0.307	0.350	0.321	0.235	0.297	0.234

Clinically pivotal ‒ the remarkably elevated NPV (0.996) demonstrates the model's exceptional precision in excluding BM among individuals categorized as low-risk: over 99 % of model-designated “BM-negative” cases truly lack metastases, substantially mitigating unwarranted patient concerns while curbing excessive utilization of subsequent neuroimaging procedures. This dual benefit simultaneously decreases healthcare expenditures and patient stress. The PR-AUC value of 0.274, while moderate, illustrates the algorithm's discriminative capability within this severely skewed dataset ‒ a characteristic hurdle in predicting rare pathological conditions. Complementing this, the clinically relevant PPV (0.137) ‒ corresponding with the disease's low prevalence and aiding in detecting a high-risk cohort meriting comprehensive diagnostic assessment ‒ equips medical practitioners with data-driven insights for optimizing surveillance protocols. These combined metrics substantiate the model's practical implementation value within clinical BM detection pathways, particularly for strategically allocating limited diagnostic resources while maintaining screening accuracy.

The classification matrix (Fig. 3E) demonstrates model efficacy through detailed performance metrics across training and evaluation datasets. During training, the matrix reveals substantial true negative classifications (12,729) alongside 4026 false positive instances, while maintaining minimal false negatives (77) against 718 accurately identified positive cases. This pattern persists in test data with 5427 true negatives, 1734 false positives, 33 false negatives, and 328 true positives, reflecting comparable classification proficiency between learned and novel data - confirming reliability across both phases. The probability distribution visualization (Fig. 3F) highlights optimal predictive performance occurring near a threshold value of 0.49, where positive and negative case densities exhibit distinct demarcation. This critical inflection point in predictive scoring corresponds to maximum discriminative capacity between diagnostic categories, facilitating precise clinical decision-making thresholds.

This analysis serves as a pivotal consideration for clinical decision-making, as it weighs accurate identification of confirmed cases against erroneous detection. The clinical utility plot (Fig. 3G) provides visual decision support through dual trajectory visualization: one curve illustrates the capture rate of true Brain Metastasis (BM) instances across varying probability cutoffs, while the complementary curve demonstrates exclusion efficacy for non-BM cases. Medical practitioners strategically select diagnostic thresholds through this framework ‒ for instance, a lower cutoff prioritizes BM case inclusion to prevent oversight, whereas a higher threshold minimizes unwarranted diagnostic procedures. This adaptive approach enhances precision in patient care protocols while optimizing resource allocation.

### Web predictor

**Fig. 4:**
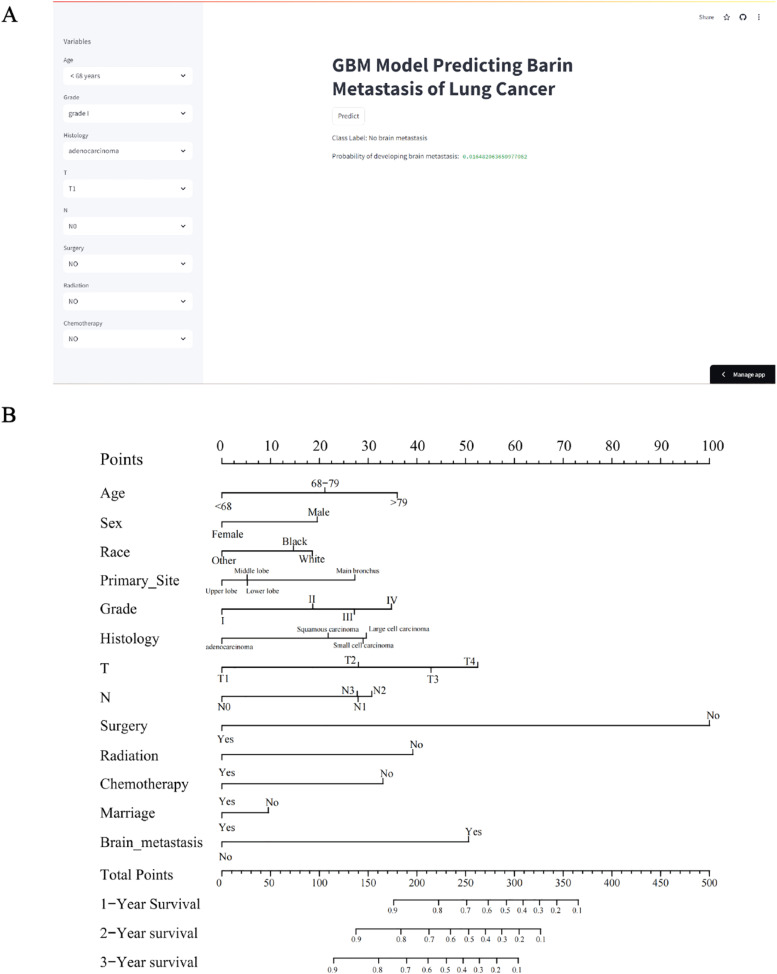
(A) A web predictor for predicting BM (brain metastasis) of lung cancer. (B) The nomogram of lung cancer patients.

Building upon the established research framework, a prognostic algorithm for cerebral metastases in pulmonary carcinoma patients was formulated through gradient boosting methodology. This computational tool demonstrated exceptional accuracy in anticipating neurological spread patterns among oncology cases. The digital assessment platform facilitates evidence-based clinical judgments by quantifying personalized metastasis risks, enabling practitioners to input demographic parameters (age, tumor grade, histopathological classification), therapeutic interventions (surgical history, radiation exposure, chemotherapeutic regimens), and TNM staging data. Upon submitting these variables to the analytical engine via the interactive portal (https://lung-cancer-brain-m-bm7dssal6atqu6qdm.streamlit.app/), healthcare professionals can select the “calculate risk” option to generate instantaneous probability estimates. The operational workflow and interface specifications are comprehensively illustrated in Fig. 4A, providing visual guidance for optimal utilization of this predictive system.

### Developing and validating of prognostic nomogram

A predictive model incorporating the previously identified 13 independent variables was developed from the training cohort to estimate lung cancer patients' Overall Survival (OS). This clinical scoring system demonstrated that surgical treatment emerged as the strongest determinant of survival outcomes, with patient age, tumor T-classification, chemotherapy administration, and cerebral metastases showing subsequent predictive importance. Survival probability projections at 12-, 24-, and 36-month intervals are visually presented in Fig. 4B For clinical application of this prognostic instrument, practitioners should begin by identifying the patient's specific clinical parameters aligned with each predictive variable (including age category, cerebral metastasis status, surgical history, and T classification) as detailed in Supplementary Table 4. Proceed by applying the standardized scoring protocol: align each clinical characteristic with its designated point value in Supplementary Table 4 (e.g., 100 points allocated for surgical intervention, 50.6 points assigned for confirmed brain metastases, 36 points corresponding to specific age brackets).

To calculate the survival prognosis score, clinicians first assign corresponding points to each prognostic factor (e.g., 34.5 points for age ≥ 79), then aggregate each component's score to obtain the total prognostic value ‒ with elevated scores indicating diminished survival prospects ‒ and subsequently apply this composite score to the nomogram for survival time estimation. The model's prognostic efficacy was assessed through three validation metrics: Concordance index (C-index), Receiver Operating Characteristic (ROC) analysis, and calibration curves. The training cohort demonstrated a C-index of 0.801 (95 % Confidence Interval [95 % CI: 0.798–0.804]), while the internal validation cohort showed comparable discriminative ability with a C-index of 0.805 (95 % CI: 0.800–0.810). Bootstrap-corrected validation in both training and internal cohorts revealed robust predictive capacity across multiple timepoints: training cohorts yielded C-indexes of 0.811 (1-year OS), 0.788 (2-year OS), and 0.774 (3-year OS), confirming the model's temporal reliability in overall survival prediction.

**Fig. 5:**
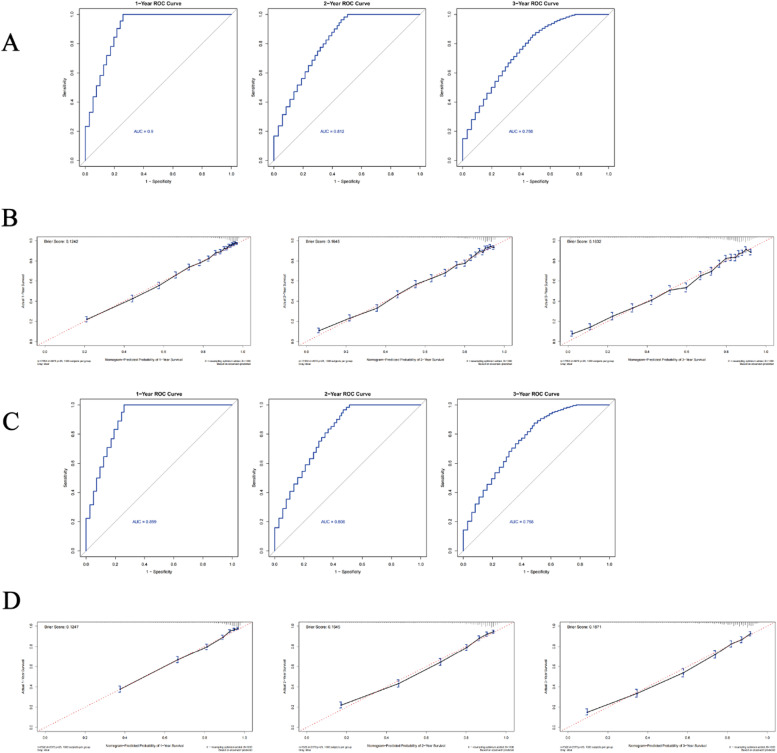
validation of the predicting nomogram. (A) 1-, 2-, and 3-year ROC (Receiver Operating Characteristic) curves of the train set. (B) 1-, 2-, and 3-year ROC (Receiver Operating Characteristic) curves of the internal test set. (C) 1-, 2-, and 3-year calibration charts of the train set. (D) 1-, 2-, and 3-year calibration charts of the internal test set.

In these analyses, the 95 % Confidence Intervals for adjusted C-index values predicting 1-year, 2-year and 3-year overall survival spanned 0.803‒0.818, 0.781‒0.794 and 0.767 - 0.780, respectively. During internal validation, bootstrap-adjusted C-index measurements for 1-, 2-, and 3-year survival outcomes reached 0.812, 0.791, and 0.780, with confidence bounds of 0.801–0.823, 0.781–0.801, and 0.770–0.790. All C-index measurements approached 0.8, demonstrating the model's robust predictive capacity for lung cancer survival outcomes across validation cohorts. ROC curve analysis revealed AUC scores of 0.9, 0.812, and 0.756 for 1-, 2-, and 3-year predictions in the training cohort (Fig. 5A), while internal testing showed comparable values of 0.899, 0.806, and 0.758 (Fig. 5C). Calibration curves exhibited favorable alignment between model predictions and observed survival rates across all time points in both training and validation datasets (Fig. 5C and 5D).

**Fig. 6:**
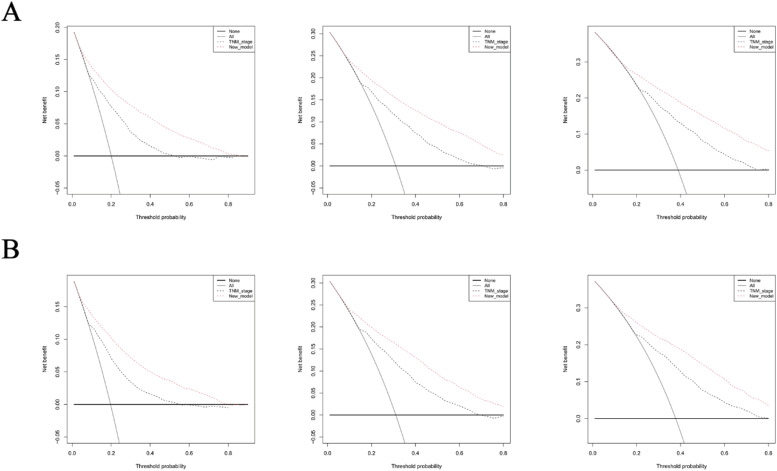
Validation of the predicting nomogram. (A) 1-, 2-, and 3-year DCA (decision curve analysis curve) curves of the train set. (B) 1-, 2-, and 3-year DCA (decision curve analysis curve) curves of the internal test set.

To evaluate the efficacy of the prognostic framework, the authors utilized Brier score metrics derived from probability calibration charts (Fig. 5B and 5D). These metrics measure how precisely predicted probabilities correspond with observed results, where diminished values reflect superior calibration performance. Both developmental and validation cohorts demonstrated concordant Brier scores for 1-, 2-, and 3-year Overall Survival (OS) forecasts, consistent with the optimal alignment visible in calibration graphics. The combined assessment ‒ incorporating both graphical calibration concordance and quantitative validation through Brier metrics ‒ confirms the present model's temporal survival estimates maintain strong correspondence with empirical outcomes. Crucially, all calibration trajectories exhibited minimal deviation from the reference 45° bisecting line. For clinical applicability verification, the authors conducted Decision Curve Analysis (DCA) contrasting the predictive algorithm against the AJCC TNM staging protocol across 1-, 2-, and 3-year. The Decision Curve Analysis (DCA) results for both training and internal validation cohorts across 1-, 2-, and 3-year intervals are presented in Fig. 6A and 6B Temporal analysis of the DCA plots revealed the innovative prognostic framework consistently outperformed the conventional AJCC TNM staging system in clinical utility throughout all observation periods, while simultaneously offering practical guidance for therapeutic strategies. Complementing these findings, the integrated discrimination improvement metrics quantitatively confirmed enhanced predictive performance: Training cohort IDI measurements progressively increased from 16.1 % (1-year) to 17.2 % (3-year OS) with statistical significance (*p* < 0.05 for all intervals), while internal validation demonstrated comparable improvements ranging from 14.9 % to 15.6 % across corresponding timepoints (all *p* < 0.05), as detailed in Fig. 6.

## Discussion

Lung cancer ranks as the second most prevalent malignancy in both genders. Current epidemiological data indicate approximately 155,000 fatalities in the United States during 2017 were linked to this disease, representing one-quarter of total cancer mortality.^20^, ^21^, ^22^ Metastatic progression accounts for the majority of lung cancer fatalities,^23^ with cerebral metastases being particularly prevalent – clinical studies reveal up to 50 % of patients eventually develop Brain Metastases (BM).^24^, ^25^, ^26^ Recent years have witnessed a marked rise in Lung Cancer Brain Metastasis (LCBM) cases, significantly compromising five-year survival rates while simultaneously straining healthcare infrastructure.^27^ This epidemiological shift stems from three interrelated causes: advancements in therapeutic interventions prolonging patient survival, enhanced diagnostic imaging enabling earlier metastasis detection, and evolving tumor biology facilitating neural tissue infiltration.

Advancements in neuroimaging modalities have enhanced the identification frequency of cerebral metastases,^28^ with demographic shifts toward older populations and socioeconomic development contributing to this epidemiological pattern.^29^^,^^30^

Therapeutic interventions and holistic care approaches for pulmonary carcinoma metastases continue to present significant clinical obstacles, requiring integrated treatment protocols. Consequently, investigating risk determinants and disease characteristics in pulmonary malignancies, coupled with precise prognostication of cerebral metastasis progression and survival outcomes, carries crucial implications for therapeutic planning within personalized oncology paradigms.

Extensive research has explored both carcinogenic elements and prognostic indicators influencing pulmonary malignancies. The pathogenesis of this disease involves complex interactions between environmental exposures and hereditary predispositions. International epidemiological investigations consistently identify tobacco use as the principal causative agent in lung cancer development.^31^ Concurrently, residential radon accumulation has emerged as the secondary etiological determinant following cigarette exposure, representing the predominant risk factor among non-smoking populations.^32^ Genomic instability and somatic mutations further contribute significantly to oncogenesis, with comprehensive molecular profiling revealing multiple driver mutations such as EGFR, ALK, ROS1, KRAS, BRAF, C-MET, RET, and HER2.^33^ These evidence-based findings regarding pulmonary carcinogenesis have gained universal recognition within clinical oncology practice.

Extensive research efforts have been dedicated to identifying prognostic indicators in lung cancer management. Research findings indicate that untreated individuals with cerebral metastases face a particularly bleak outlook, surviving an average of 1‒2 months post-diagnosis. Clinical evidence demonstrates that Whole-Brain Radiation Therapy (WBRT) extends median survival to 4‒6 months, solidifying its position as the primary treatment approach for multiple brain metastases cases.^34^^,^^35^ Cytotoxic drug regimens substantially impact clinical outcomes in metastatic patients, with platinum-based combinations historically serving as the cornerstone therapy for advanced NSCLC. Despite therapeutic advancements, metastatic NSCLC maintains a discouraging 5-year survival rate of approximately 7 %.^36^ Surgical intervention studies by Bryan and Donington revealed improved survival outcomes and localized disease control when integrated into multimodality treatment plans for operable stage IIIA malignancies.^37^

Previous research by Chati et al. established a predictive nomogram framework for advanced non-small cell lung cancer patients, incorporating variables such as demographic characteristics, tumor staging parameters, and therapeutic approaches.^38^ Subsequent investigations by Li Z and Mu X expanded this methodology to prognostic modeling in small cell and large cell carcinomas.^39^^,^^40^ Current research in this field presents two primary constraints: insufficient utilization of large-scale datasets for lung cancer prognostic analysis, coupled with methodological limitations in existing models that have overlooked the integration of both NSCLC and SCLC subtypes. The present investigation addresses these gaps by incorporating four major histological subtypes (adenocarcinoma, squamous cell carcinoma, SCLC, and LCLC) concurrently within the analytical framework while accounting for metastatic progression, thereby enhancing prognostic prediction comprehensiveness.

Nevertheless, scholarly publications demonstrate a notable scarcity of studies examining BM in terminal-phase pulmonary carcinoma. Contemporary explorations within this field mainly emphasize two crucial dimensions. Initially, insufficient attention has been directed toward comprehensive investigation of elevated-risk prognostic indicators correlated with cerebral metastases in lung malignancies. Moreover, the dynamic interactions between these autonomous predictive variables remain insufficiently mapped. Subsequently, despite artificial intelligence and extensive data processing demonstrating remarkable analytical capacities with substantial progress in recent years, structured explorations of cerebral metastasis prediction systems utilizing AI methodologies in lung cancer remain underrepresented. Therefore, integrative studies addressing these gaps are critically imperative to improve prognostic evaluation and metastatic pattern anticipation in pulmonary oncology.

By employing comprehensive data analysis from the SEER registry, this investigation utilized logistic regression modeling to pinpoint significant predictors of cerebral metastasis. Thirteen clinically relevant parameters spanning demographic characteristics, tumor biology, and therapeutic interventions were analyzed, including patient age, gender, ethnicity, tumor origin, laterality, differentiation status, histological subtype, tumor extent (T-stage), nodal involvement (N-stage), surgical intervention, radiotherapy administration, systemic treatment history, and marital status. Inter-variable associations were visualized through Spearman's rank correlation heatmaps, with Fig. 2A demonstrating minimal collinearity among the selected variables. Multivariate analysis identified eight clinically significant predictors of cerebral dissemination after adjusting for potential confounders: patient age at diagnosis, tumor histopathology, differentiation grade, primary tumor extent, regional lymph node involvement, surgical resection status, radiation therapy implementation, and chemotherapy administration history. The development of predictive frameworks for cerebral metastasis in progressive pulmonary malignancies represents a critical advancement comparable in importance to the identification of these prognostic determinants.

Within this analytical framework, six predictive models were developed using machine learning techniques. An internal validation process employing a 10-fold cross-validation approach was implemented (Fig. 3A), through which the Gradient Boosting Machine (GBM) algorithm demonstrated superior predictive capability compared to other candidates. Building upon these findings, the authors developed a publicly accessible web-based prediction platform utilizing the GBM architecture (https://lung-cancer-brain-m- bm7dssal6atqu6qdmung2h.streamlit.app/). This computational system allows precise estimation of cerebral metastasis probability through comprehensive clinical parameter analysis. Medical practitioners can utilize the web-based interface to input patient-specific clinical data and obtain instant risk stratification results, thereby enhancing therapeutic planning and clinical management strategies.

Subsequently, Cox proportional hazards regression modeling was utilized to pinpoint 13 distinct prognostic indicators linked to pulmonary carcinoma outcomes, while simultaneously developing time-dependent survival probability estimates spanning 1-, 2-, and 3-year intervals. The graphical nomogram facilitates simultaneous incorporation of diverse clinical parameters through its visual analytics framework, offering an integrated predictive system for customized survival projections. This multidimensional assessment methodology empowers clinicians to perform risk stratification with enhanced precision based on patients' biological profiles, thereby enabling optimized therapeutic regimen customization. Such granular prognostic insights significantly improve collaborative treatment planning processes between medical teams and affected individuals, fostering evidence-based clinical interventions that elevate the quality of oncological care delivery.^41^^,^^42^

This study boasts several prominent strengths. First, a machine-driven statistical model was developed to forecast brain metastasis in lung cancer patients. To the best of our knowledge, this represents the inaugural effort to leverage machine learning for constructing a predictive model of lung cancer brain metastasis, thereby expanding the understanding of advanced lung cancer. Second, the study further probed the associations among independent high-risk factors, offering novel avenues for future clinical research. Specifically, clinical research ought not only to examine the occurrence of metastasis in patients but also account for the relationships between distinct high-risk factors, and gain a deeper comprehension of their mutual influences. This would help to further mitigate adverse factors potentially inducing metastasis during the perioperative phase. Additionally, the authors have crafted not only a predictive model for lung cancer brain metastasis but also a nomogram for prognosticating lung cancer survival time. The integration of these two models can substantially aid clinicians in making more well-informed decisions for lung cancer patients. Fourth, considering the recent release of the ninth edition of the AJCC lung cancer staging system, the clinical data employed this ninth edition TNM staging system, facilitating more remarkable outcomes.

### Limitation

Nevertheless, this investigation presents inherent methodological constraints that warrant recognition. While advancing the field, the research encounters two primary limitations. Current machine learning methodologies predominantly utilize statistical approaches or opaque model architectures,^43^ introducing significant theoretical limitations in predictive accuracy. This opacity restricts the mechanistic interpretation of critical prognostic factors (including surgical interventions and tumor staging) in cerebral metastasis development, complicating clinical implementation of predictive analytics. Additionally, being conducted at a single institution, the research features a geographically constrained participant pool that may limit demographic diversity and generalizability of findings.

The utilization of machine learning algorithms on large-scale datasets may enhance predictive accuracy.^44^ The validation protocol relied solely on internal evaluations within the SEER database (partitioned into training and internal validation cohorts), lacking external verification through independent multicenter studies ‒ a limitation that affects the broader applicability of both the GBM algorithm and prognostic nomogram. Fourthly, potential temporal confounding arises from the SEER registry's absence of time-stamped clinical records. The chronological relationship between therapeutic interventions (surgical procedures, radiotherapy, and chemotherapy) and subsequent BM diagnosis could not be definitively established, potentially introducing measurement bias when considering these treatments as baseline predictors, despite adherence to SEER methodological standards. Fifthly, critical clinical variables such as ECOG performance scores, detailed smoking history metrics (including pack-year calculations), and molecular profiling data (EGFR/ALK mutation status) were excluded due to database limitations, constraining the model's clinical specificity. Furthermore, the age stratification threshold was determined algorithmically using X-tile software rather than being grounded in established clinical parameters.

The absence of robust theoretical foundations and potentially undermining consistency among different cohorts represent key limitations. Sixth, the omission of cases containing “unknown” predictor variables (such as AJCC stages and histology) in complete-case analytical approaches could introduce selection bias, as patients with missing data might constitute distinct clinical subgroups not fully representative of real-world clinical scenarios. Subsequent investigations will tackle these limitations through multicenter external validation studies, incorporating temporally annotated data streams, and integration of previously unaccounted clinical parameters to strengthen predictive model reliability.

## Conclusion

Consequently, this research developed a predictive algorithm using machine learning techniques to assess cerebral metastasis risks in lung cancer patients, incorporating eight routinely available clinical-pathological parameters from standard medical practice. Among various models evaluated, the Gradient Boosting Machine exhibited superior predictive accuracy. This optimized GBM framework demonstrates clinical utility for anticipating neurological metastases, enabling physicians to enhance precision in therapeutic decision-making for pulmonary carcinoma cases. Additionally, investigators created a comprehensive prognostic scoring system through big data analytics. The integrated application of these predictive tools facilitates simultaneous assessment of metastasis probability and survival outcomes, providing multidimensional clinical insights for patient management.

## Ethics approval and consent to participate

Not applicable. The authors confirm that no experimental procedures involving humans or animals were conducted during this research.

## Consent for publication

All authors take full responsibility for ensuring the validity and integrity of this work. Any issues concerning accuracy or ethical standards have been thoroughly examined and addressed.

## Availability of data and materials

The research data supporting this study are openly accessible through the SEER database (https://seer.cancer.gov). Specific configurations used in SEER*Stat analyses, computational scripts, or additional methodological details may be requested from the corresponding author. Full technical documentation and analytical resources will be made available to facilitate replication studies.

## Funding

China-Japan Union Hospital of Jilin University MED+XInterdisciplinary Discipline 10.13039/501100013047Cultivation Program No 2025022. Clinical Comparative Study of Single-port thoracoscopic radical resection of lung Cancer and traditional thoracoscopic radical resection of 10.13039/100003089lung cancer (No.SCZSY201611). The Special Project for Health Research Talents of Jilin Province [construction of 3D model for precise navigation of thoracoscopic lung segmental (subsegmental) resection], funded by the Jilin 10.13039/501100011791Provincial Department of Finance (No. 2023SCZ27). Research on the prediction model of invasive Adenocarcinoma of the lung based on multimodal deep learning of clinical-radiomics-genomics (No. YXJL-2022-0080-0443).

## Data availability

The datasets generated and/or analyzed during the current study are available from the corresponding author upon reasonable request.

## CRediT authorship contribution statement

**Yukai Zeng:** Visualization. **Fengwu Lin:** Funding acquisition. **Lening Zhang:** Supervision. **Jingyuan Jiang:** Writing – review & editing. **Chen Zhang:** Validation. **Dongliang Qin:** Data curation. **Zhe Zhang:** Writing – original draft.

## Declaration of competing interest

The authors declare no conflicts of interest.

## Appendix Supplementary materials

Image, application 1
