# Supplementary material for: Development and validation of a machine learning model and nomogram for predicting brain metastasis in lung cancer: a population-based study
Source: Clinics (Sao Paulo). 2025 Nov 13;80:100843. doi: 10.1016/j.clinsp.2025.100843 (PMC12662121; doi:10.1016/j.clinsp.2025.100843)
Supplement: Supplementary file 1 [file mmc1.docx]

CLINICS-D-25-01138_Supplementary Material

**Supplementary Table 1** The Chi-Square test of train set and internal test set.

| **Variables** | **p-value for Chi-Square test** |
| --- | --- |
| Age | 0.258 |
| Sex | 0.738 |
| Race | 0.711 |
| Primary Site | 0.730 |
| Laterality | 0.156 |
| Grade | 0.564 |
| Histology | 0.796 |
| T | 0.318 |
| N | 0.508 |
| Surgery | 0.859 |
| Radiation | 0.277 |
| Chemotherapy | 0.683 |
| Marriage | 0.132 |
| Brain metastasis | 0.357 |

**Supplementary Table 2** Full list of candidate predictors.

| **Category** | **Candidate Predictor** | **Definition/Classification** |
| --- | --- | --- |
| Demographic | Age | Continuous; cut-off via X-tile 3.6.1 (Section 2.2) |
|  | Sex | Binary: Male/Female |
|  | Race | Categorical: White/Black/Other (SEER coding) |
|  | Marital Status | Categorical: Married/Single/Divorced/Widowed |
| Tumor-Related | Primary Site | Lung and Bronchus (ICD-O-3/WHO 2008: “Lung and Bronchus”) |
|  | Laterality | Categorical: Left/Right/Bilateral |
|  | Histology | Categorical: Adenocarcinoma (8140/3), Squamous cell carcinoma (8070/3), Large cell carcinoma (8012/3), Small cell lung cancer (8041/3) (ICD-O-3) |
|  | Grade | Categorical: Well/Moderately/Poorly/Undifferentiated |
|  | T Stage | AJCC 9^th^ edition (T1–T4; recoded from AJCC 7^th^ edition, SEER standards) |
|  | N Stage | AJCC 9^th^ edition (N0–N3; recoded from AJCC 7^th^ edition, SEER standards) |
| Treatment-Related | Surgery | Binary: Yes/No |
|  | Radiation | Binary: Yes/No |
|  | Chemotherapy | Binary: Yes/No |
| Outcome-Related | Brain Metastasis | Binary: Baseline status (Yes/No at lung cancer diagnosis) |

**Supplementary Table 3** Default parameters of machine learning algorithms.

| **Algorithm** | **Library Default Parameters** |
| --- | --- |
| Random Forest (RF) | n_estimators=100, criterion='gini', max_depth=None, min_samples_split=2, min_samples_leaf=1, max_features='sqrt' |
| XGBoost (XGB) | objective='binary:logistic', learning_rate=0.3, max_depth=6, n_estimators=100, subsample=1.0, colsample_bytree=1.0 |
| Gradient Boosting (GBM) | loss='log_loss', learning_rate=0.1, n_estimators=100, max_depth=3, min_samples_split=2, min_samples_leaf=1 |
| Logistic Regression (LR) | penalty='l2',  C=1.0,  solver='lbfgs',  max_iter=100, multi_class='auto', fit_intercept=True |
| Decision Tree (DT) | criterion='gini', max_depth=None, min_samples_split=2, min_samples_leaf=1, max_features=None |
| Gaussian NB (NBC) | priors=None, var_smoothing=1e-09 |

**Supplementary Table 4** Default parameters of machine learning algorithms.

| **Variable** | **Points** |
| --- | --- |
| Age ≥ 79 | 36.0 |
| Age = 68‒79 | 21.1 |
| Brain_metastasis = Yes | 50.6 |
| Chemotherapy = Yes | 33.0 |
| Grade II | 18.7 |
| Grade III | 27.2 |
| Grade IV | 34.8 |
| Histology = Large cell carcinoma | 29.6 |
| Histology = Small cell carcinoma | 29.0 |
| Histology = Squamous carcinoma | 21.8 |
| Marriage = Yes | 9.5 |
| N = N1 | 27.9 |
| N = N2 | 30.8 |
| N = N3 | 27.8 |
| Primary_Site = Lower lobe | 5.2 |
| Primary_Site = Main bronchus | 27.3 |
| Primary_Site = Middle lobe | 5.2 |
| Race = Black | 3.9 |
| Race = Other | 18.5 |
| Radiation = Yes | 39.2 |
| Sex = Male | 19.5 |
| Surgery = Yes | 100.0 |
| T = T2 | 28.0 |
| T = T3 | 42.9 |
| T = T4 | 52.5 |
